# Vacuum-assisted core biopsy of the breast

**DOI:** 10.1186/bcr2967

**Published:** 2011-11-04

**Authors:** S Saikia, L Lunt

**Affiliations:** 1 Queen Elizabeth Hospital, Gateshead, UK

## Introduction

Vacuum-assisted core biopsy (VACB) is a highly effective method of sampling breast tissue. Breast care nurses, at the Queen Elizabeth Breast Unit in Gateshead, raised concerns about pain experienced by patients during this procedure. The aim of this study was to assess how patients perceive VACB.

## Methods

Prospective data collection for 6 months from November 2010 of consecutive patients undergoing VACB. Data were collected using a questionnaire about pain experienced immediately and 4 weeks post procedure, using the Numerical Rating Scale and Short Form McGill validated pain scores [1].

## Results

Fifty questionnaires were completed. Immediately post procedure, 88% felt no or mild pain. Of those reporting pain, 100% had a sensory dimension. Four weeks later the memory of the pain experienced during the procedure was worse in 46%. In this group, pain 4 weeks post procedure had an affective dimension in 50% of cases. Four weeks post procedure, 91% felt no or mild pain. One hundred per cent would reassure a friend about the procedure.

## Conclusion

Minimal pain was experienced immediately or 4 weeks post procedure. There is a discrepancy between pain experienced during the procedure compared with the memory of it. The effect of the biopsy result could be a contributory factor. Further work linking responses to histology may be revealing.
